# Does Quinine Affect the Hearing?

**Published:** 1879-01

**Authors:** 


Does Quinine Affect the Hearing?—The belief is general among the laity that the prolonged use of quinine affects the hearing. Medical men have generally disbelieved this, and attributed the notion to prejudice. Dr. Roosa, of New York, has been examining the evidence, such as he can procure, and is inclined to believe that in some cases there is a permanent nervous affection of the ear produced, which justifies the opinion of the laity.—*Med. and Surg. Rep.*

